# Overactive mTOR signaling leads to endometrial hyperplasia in aged women and mice

**DOI:** 10.18632/oncotarget.13919

**Published:** 2016-12-12

**Authors:** Preety Bajwa, Sarah Nielsen, Janine M. Lombard, Loui Rassam, Pravin Nahar, Bo R. Rueda, J. Erby Wilkinson, Richard A. Miller, Pradeep S Tanwar

**Affiliations:** ^1^ Gynaecology Oncology Group, School of Biomedical Sciences and Pharmacy, Callaghan, New South Wales, Australia; ^2^ Hunter Cancer Biobank, Callaghan, New South Wales, Australia; ^3^ School of Medicine and Public Health, University of Newcastle, Callaghan, New South Wales, Australia; ^4^ Department of Medical Oncology, Gynaecology Oncology, Calvary Mater Newcastle, Waratah, New South Wales, Australia; ^5^ Gynaecology and Obstetrics, John Hunter Hospital, New Lambton, New South Wales, Australia; ^6^ Vincent Department of Obstetrics and Gynecology and Department of Obstetrics & Gynecology, Vincent Center for Reproductive Biology, Massachusetts General Hospital and Gynecologic Oncology Division, Massachusetts General Hospital, Harvard Medical School, Boston, MA, USA; ^7^ Department of Pathology, Unit for Laboratory Animal Medicine, University of Michigan School of Medicine, Ann Arbor, MI, USA; ^8^ Department of Pathology and Geriatrics Center, University of Michigan, Ann Arbor, MI, USA

**Keywords:** endometrial, mTOR, rapalogs, aging, PI3K, Pten, Gerotarget

## Abstract

During aging, uncontrolled epithelial cell proliferation in the uterus results in endometrial hyperplasia and/or cancer development. The mTOR signaling pathway is one of the major regulators of aging as suppression of this pathway prolongs lifespan in model organisms. Genetic alterations in this pathway via mutations and/or amplifications are often encountered in endometrial cancers. However, the exact contribution of mTOR signaling and uterine aging to endometrial pathologies is currently unclear. This study examined the role of mTOR signaling in uterine aging and its implications in the development of endometrial hyperplasia. The hyperplastic endometrium of both postmenopausal women and aged mice exhibited elevated mTOR activity as seen with increased expression of the pS6 protein. Analysis of uteri from *Pten* heterozygous and *Pten* overexpressing mice further confirmed that over-activation of mTOR signaling leads to endometrial hyperplasia. Pharmacological inhibition of mTOR signaling using rapamycin treatment suppressed endometrial hyperplasia in aged mice. Furthermore, treatment with mTOR inhibitors reduced colony size and proliferation of a PTEN negative endometrial cancer cell line in 3D culture. Collectively, this study suggests that hyperactivation of the mTOR pathway is involved in the development of endometrial hyperplasia in aged women and mice.

## INTRODUCTION

The mammalian uterus is one of the most regenerative organs that undergoes the cyclical process of degeneration and regeneration during each oestrous cycle [1]. The uterus plays a critical role in pregnancy and child birth. The mammalian uterus is divided into three compartments, namely endometrium (luminal and glandular epithelial cells), stroma, and myometrium (smooth muscle cells) [1]. After fertilization of an egg in the fallopian tube, the resulting embryo moves to the uterine cavity where its interaction with the receptive endometrium initiate a cascade of events leading to implantation, decidualization, and the establishment of pregnancy. Any aberrations during this process lead to premature termination of pregnancy and abortion [2].

Age is a major risk factor for many diseases, including infertility and cancer. Advanced maternal age is strongly associated with various foetal and obstetric complications. The risk of abortion, ectopic pregnancy, preterm labour, intrauterine growth restriction or death, and congenital defects is significantly increased in women older than 45 years of age [3]. Studies using human uterine samples and animal models have suggested that age-related changes in the uterus contribute to fertility defects [4]. Treatment of young and aged mice with ovarian hormones showed that aged uteri failed to undergo normal decidualization and presented with abnormally high proliferation of endometrial cells [5, 6]. Examination of mice and pig uteri revealed that a higher number of degenerated embryos are present at the implantation sites in aged uteri suggesting that the inability of aged uteri to carry the implanted embryo to term contributes to the age-related decline in fecundity [7]. This view is further supported by the outcomes of a study in the Cheetah (*Acinonyx jubatus*) examining causes of the high prevalence of subfertility in this species [8]. Analysis of Cheetah female reproductive tracts showed normal ovarian function with fertilizable oocytes in both young and aged females. However, histological examination of the uteri revealed endometrial hyperplasia in 87% of aged females [8]. Collectively, these studies suggest that age-related changes in the uterus can impair female fertility in multiple species.

A significant proportion (~24%) of perimenopausal or postmenopausal women develop endometrial hyperplasia with age, and up to 28% of these women go on to develop endometrial cancer [9, 10]. Endometrial cancer is the most common gynaecological cancer that affects 757,190 women in the USA, and is primarily a disease of aged women with median age of diagnosis at 62 years [11]. Given that many women of the modern world are intentionally delaying child bearing because of competing demands of better education and career, and because fertility rates are falling in developed countries, it is important to understand the contributions of uterine aging to fertility defects and endometrial hyperplasia/cancer. Previously, we and others have shown that mTOR (mammalian target of rapamycin) is a key regulator of aging, and pharmacological or genetic suppression of this pathway extends healthy lifespan in many species [12–18]. Our earlier work has established that overactive mTOR drives the growth of endometrial cancer [19, 20]. However, it is currently unclear if age related changes in mTOR activity contribute to the development of uterine pathologies, such as endometrial hyperplasia, in aged women. In this study, using human tissue samples, endometrial cells, and mouse models, we have established that hyperactive mTOR signaling involved in dysregulated growth of uterine epithelial cells in aged women and mice.

## RESULTS

### Increased mTOR signaling in hyperplastic endometrium of aged human and mouse

**Figure 1:**
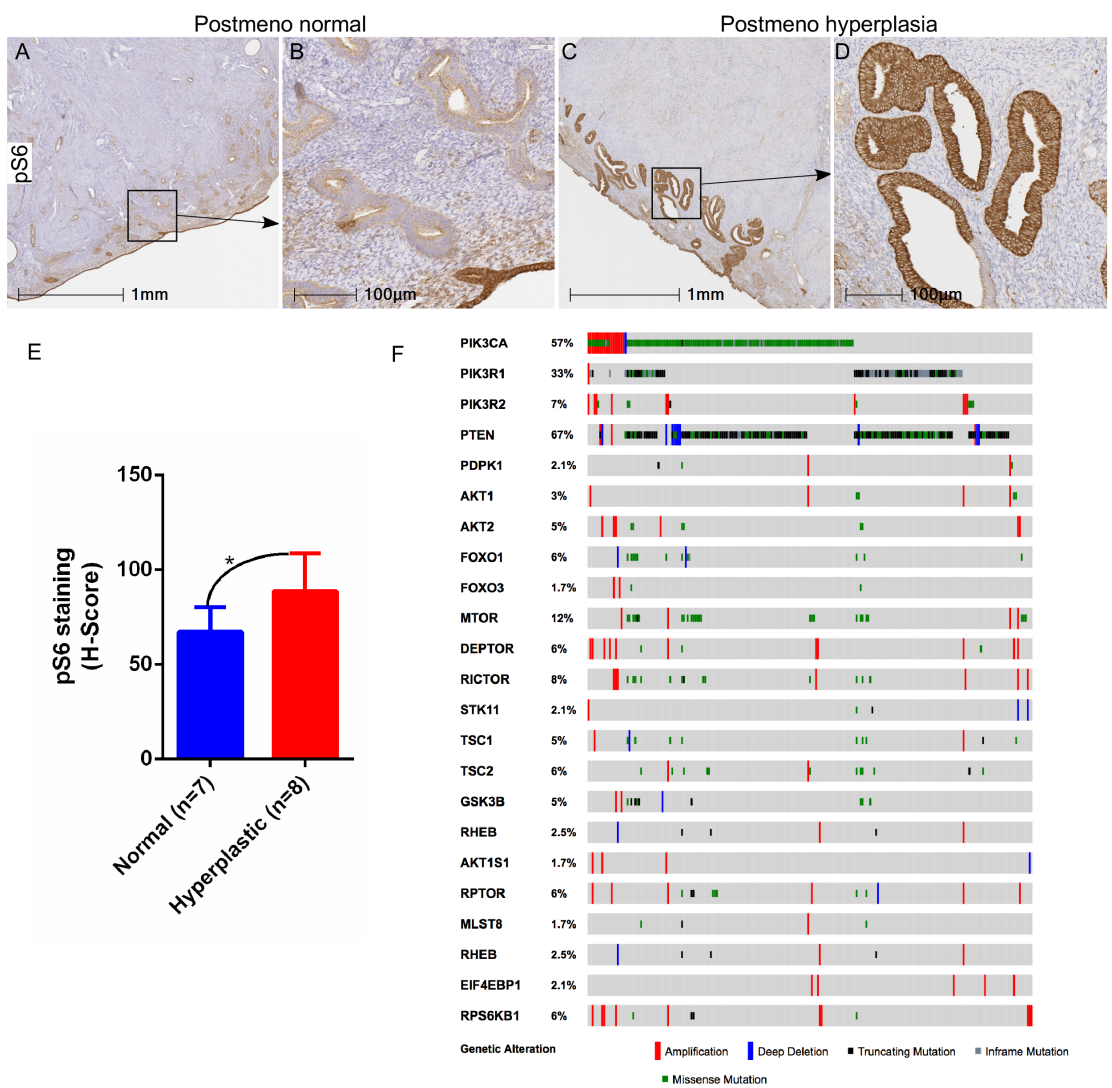
Hyperactive mTOR signaling in human endometrial hyperplasia and cancer Increased expression of pS6, a marker for mTOR activation, was observed in hyperplastic post-menopausal human endometrium compared to normal post-menopausal endometrium **A**.-**D**. Panel B and D is a higher magnification image of boxed area in panel A and C, respectively. H-score quantification of pS6 staining performed using Halo^™^ image analysis software **E**. TCGA dataset analysis for endometrial cancer showed alterations in components of the PI3K-mTOR pathway **F**. *P < 0.05, Student's t-test.

To examine the involvement of mTOR signaling in the development of endometrial hyperplasia in aged women, we analysed the expression of the phosphorylated form of S6 ribosomal protein (pS6), a well-known marker for mTOR activation, in both normal (*N* = 7) and hyperplastic (*N* = 8) endometrium, collected from post-menopausal women. Compared to the normal post-menopausal endometrium (Figure 1A and 1B), increased pS6 protein expression was observed in abnormal epithelial glands present in the hyperplastic post-menopausal endometrium (Figure 1C and 1D). Using the area quantification algorithm for pixel intensities, we calculated the H-score for pS6 staining and found significantly a higher H-score for hyperplastic post-menopausal endometrium as compared to normal (Figure 1E). Further, we examined The Cancer Genome Atlas (TCGA) for endometrial cancer and found genetic alterations in 95% (229/242) of patients in several key components of the PI3K-mTOR pathway, including PI3KCA (57%), PTEN (67%), PIK3R1 (33%) and mTOR (12%) (Figure 1F).

**Figure 2:**
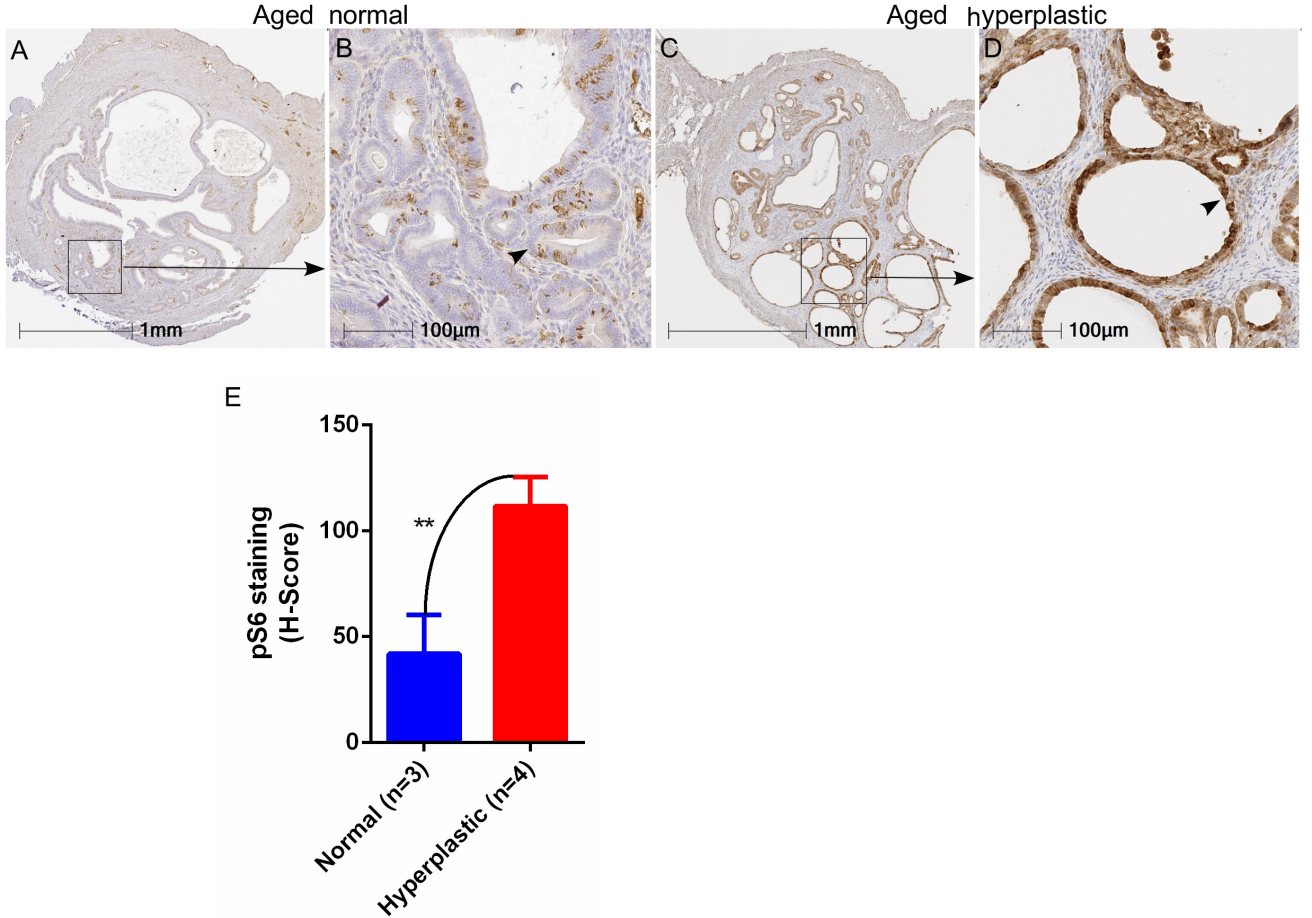
Heightened mTOR signaling in hyperplastic uteri of aged mice Immunostaining for pS6 marker in normal and hyperplastic aged uteri **A**.-**D**. Enhanced expression of pS6 was observed in endometrial cysts (marked with an arrowhead in panel D) of hyperplastic uteri of aged mice. Graph showing H-score of intensity for pS6 staining **E**. **P < 0.01, Student's t-test.

Similar to women, aged mice can be afflicted by endometrial hyperplasia and/or cancer [21]. To confirm whether hyperactive mTOR signalling is also associated with the development of hyperplastic lesions in the uterus of aged mice, we performed immunostaining of pS6, a marker of mTOR activity, on normal (*N* = 3) and hyperplastic (*N* = 4) uteri collected from aged mice (18-20 months old). As was the case for human patients, elevated expression of the pS6 protein was observed in hyperplastic uteri of aged mice especially in the enlarged cystic glands (Figure 2C-2E), whereas normal expression of pS6 was characteristic of endometrial cells in uteri that did not show hyperplasia (Figure 2A and 2B). The H-score for quantification of the intensity of pS6 staining also confirmed a significant increase in hyperplastic uteri as compared to the aged controls (Figure 2E). Collectively, these data showed that hyperactivation of mTOR signaling occurs in endometrial pathologies observed in aged mice and women.

### mTOR signaling controls the hyperplastic growth of uterus

**Figure 3:**
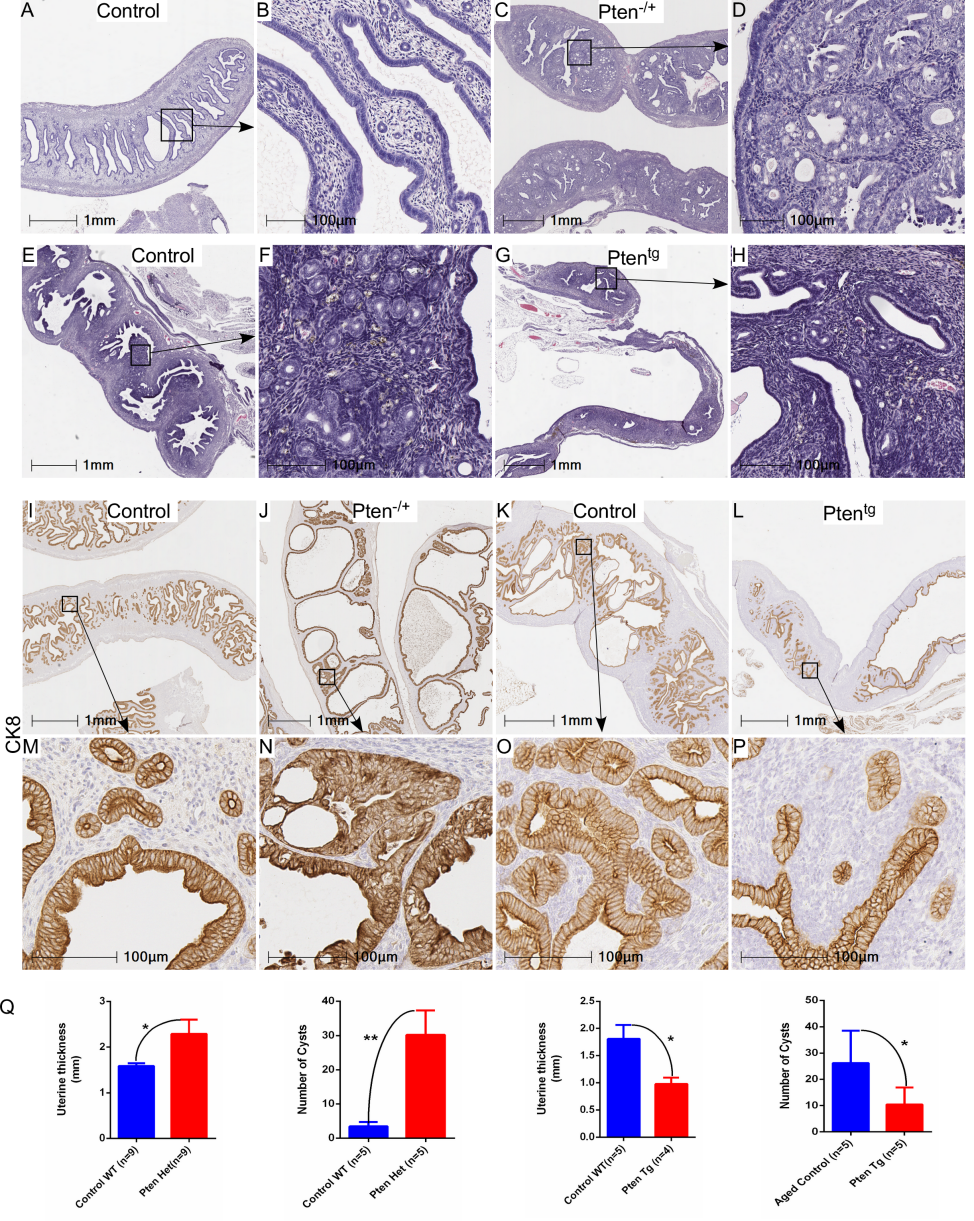
Genetic alterations in Pten, a negative regulator of mTOR signaling, contribute to the hyperplastic growth of uterus Haematoxylin and eosin-stained sections of age-matched control and Pten^+/−^ uteri **A**.-**D**. B and D represent higher magnification images of boxed areas in panel A and C, respectively. Abnormal hyperplastic growth was observed in aged control uteri, which was not present in age matched Pten^tg^ uteri **E**.-**H**. Boxed areas in panel E and G are presented at higher magnification in panel F and G, respectively. CK8, a marker for epithelial cells, staining in control **I**. and **M**. Pten^+/−^ **J**. and **N**., aged uteri **K**. and **O**. and Pten^tg^ uteri L. and P. M, N, O, and P represent higher magnification images of boxed areas in I, J, K and L, respectively. Q. Graphs showing quantification of uterine thickness and number of cysts in Pten^+/−^ and Pten^Tg^ mice as compared to their respective controls. *P < 0.05, **P < 0.01, Student's t-test.

Significant genetic alterations in the PI3K-mTOR pathway are observed in human endometrial cancer (Figure 1F) and the loss of *Pten* or overactivation of mTOR signaling results in the development of similar tumours in mouse models [19, 22]. To understand if modulation in the level of mTOR signaling will affect age-associated endometrial hyperplasia in mice, we utilised two well established mouse models, *Pten* overexpressing (Pten^tg^) and *Pten* heterozygous (Pten^-/+^) mice [23, 24]. We collected uteri from aged *Pten* heterozygous (Pten^+/−^, *N* = 9/each; age 7-8 months), *Pten* transgenic (Pten^tg^, *N* = 5/each; age: 26-27 months) and their age-matched wild type (WT) control mice. Histological examination of uteri from Pten^+/−^ mice revealed abnormal glandular architecture and hyperplastic epithelial growths (Figure 3C-3D). In comparison, normally distributed round endometrial glands were present in age-matched wild type control mice (Figure 3A and 3B). In contrast to Pten^+/−^ mice, uteri of aged Pten^tg^ (26-27 months old) mice had a normal endometrial epithelial lining and glandular arrangement (Figure 3G-3H), which was similar to that seen in relatively young wild type mice (Figure 3A and 3B). As expected, abnormal glandular expansion and crowding with much less intervening stroma indicative of hyperplasia, was observed in aged WT control uteri (Figure 3E-3F; 26-27 months old). Immunolocalization of CK8, a marker of epithelial cells, further confirmed phenotypic changes in the uteri of Pten^+/−^, Pten^tg^, compared to their respective control mice (Figure 3I-3P). Quantification of uterine thickness and number of epithelial cysts revealed significant increase in the Pten^+/−^ mice compared to their wild type controls (Figure 3Q). An opposite trend was observed in the Pten^tg^ mice (Figure 3Q).

**Figure 4:**
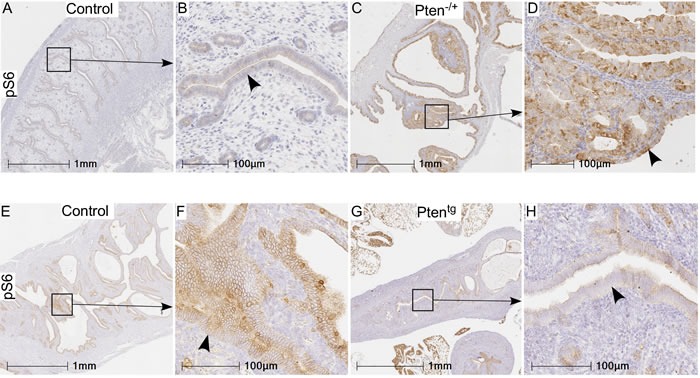
pS6 protein expression in uteri of Pten **^+/−^** and Pten**^tg^** mice as compared to their age-matched controls. Increased expression of pS6 was observed in hyperplastic uteri (arrowhead in panel D) of Pten^+/−^ mice compared to controls (age: 7-8 months) **A**.-**D**. Relative to controls **E**. and **F**. (age: 26-27 months), weak or no expression of pS6 was present in Pten^tg^ mice **G**.-**H**. Panel B, D, F, and H are higher magnification images of boxed areas in panel A, C, E, and G, respectively.

In order to confirm that the mTOR signaling differs in Pten^+/−^ and Pten^tg^ mice, we performed immunohistochemical localization of pS6 in uteri of Pten^+/−^ and Pten^tg^ mice. As expected, there was increased expression of pS6 in the hyperplastic uteri of the Pten^+/−^mice as compared to normal WT controls (Figure 4A-4D). In contrast, decreased expression of pS6 was present in the Pten^tg^ mice (Figure 4E-4H). In summary, these results have established that the loss of *Pten* is responsible for the development of hyperplastic changes in the uterus and its overexpression is sufficient to inhibit these pathological changes in aged uteri.

### Pharmacological inhibition of mTOR signaling suppresses age-associated pathological changes in uterus

**Figure 5:**
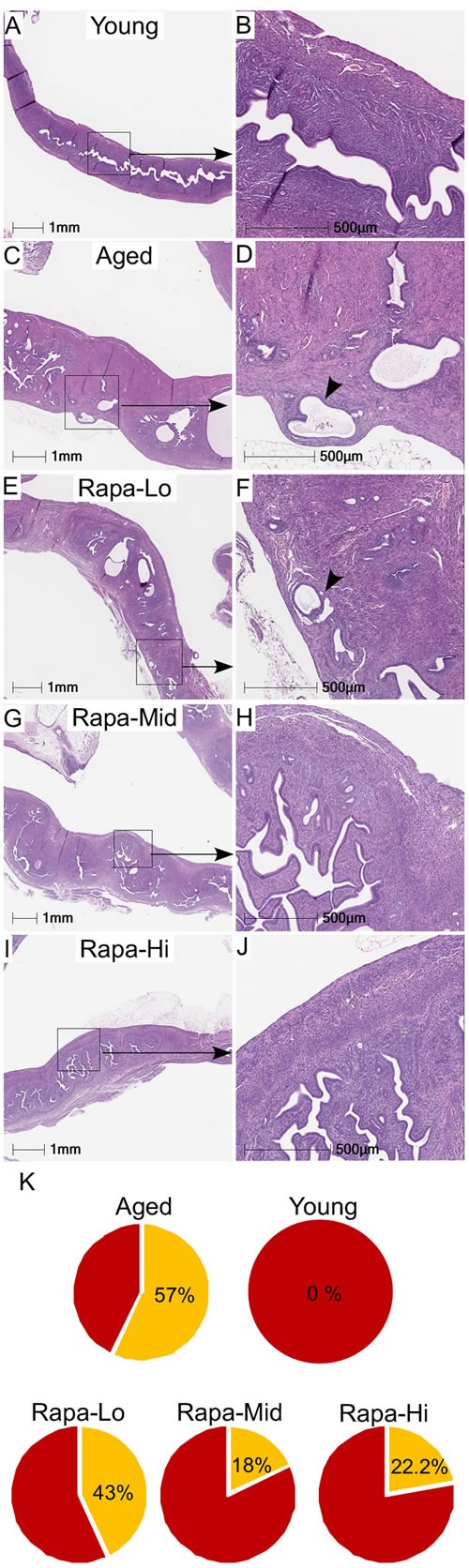
Chronic rapamycin treatment suppresses age-associated hyperplastic changes in the mouse uterus Histology of uterus of the young mice showing normal morphology and endometrial glands **A**. and **B**. Representative H&E-stained sections of aged control mice showed the presence of endometrial glands into the myometrium (shown with an arrowhead in panel D) **C**. and **D**. Treatment with rapamycin reduced the presence of the age-associated abnormal epithelial lesions in uteri of the aged mice to varying percentages with different doses **E**.-**K**.

To evaluate if the inhibition of mTOR signaling using an mTOR inhibitor will suppress the pathological changes in the aged uterus, we collected uteri from mice that were treated with rapamycin from 9 months of age at three different doses (4.7, 14, or 42 parts per million in food) for 13 months. Previously, we have shown that chronic rapamycin treatment significantly suppressed the age-associated hyperplastic changes in ovarian surface epithelium and endometrium [14, 23]. In agreement with our previous observations [14], assessment of cystic glands in uteri showed a significant reduction in the cystic endometrial hyperplasia of aged mice uteri treated with a high dose of rapamycin, reducing this to levels comparable to those of uteri of young mice (SFigure 1). Neither of the two lower rapamycin doses resulted in a significant diminution in the age-related occurrence of cystic endometrial hyperplasia (SFigure 1). Abnormal localization of endometrial glands in myometrium (smooth muscle) occurs in both human and animal pathologies such as adenomyosis and endometrial cancer [25, 26]. We observed endometrial glands in the myometrium of 57% (*N* = 4/7) of aged mice, but not in young mice (Figure 5A-5D). Rapamycin treatment reduced the incidence of these lesions to 43% (*N* = 3/7) mice with the low dose, 22% (*N* = 2/9; *p* = 0.15) mice with the high dose, and 18% (2/11; *p* = 0.09) mice with the medium dose (Figure 5E-5K). So while there is a trend towards lower lesion incidence in the Rapa mice, it is not statistically significant. Overall, these results showed that chronic suppression of mTOR signaling inhibits age-related incidences of endometrial hyperplasia and adenomyosis in mice.

### mTOR inhibitors suppress the growth of 3D spheroids of endometrial cancer cells

To test if pharmacological suppression of mTOR signaling will suppress the growth of endometrial cancer, we treated cells of an endometrial line, Ishikawa, with two FDA-approved mTOR inhibitors (Everolimus and BEZ235). We chose Ishikawa cells because these cells harbor inactivating mutations (V317fs; V290fs) in the *PTEN* gene [27]. These cells were cultured as 3D spheroids in matrigel to mimic endometrial morphology and were treated with increasing doses of mTOR inhibitors (SFigure 2). Treatment with Everolimus or BEZ235 significantly decreased cell viability of these cells in a dose dependent manner (SFigure 2B and C). In order to assess the effect of these inhibitors on cell growth, colony size was monitored at regular intervals (24h, 48h and 72h) after exposure to mTOR inhibitors. There was a decrease in the size of spheroids with increasing doses of inhibitors (SFigure 2D and E). To confirm the efficacy of these inhibitors in suppressing mTOR activity, we performed AKT/mTOR protein arrays and showed that treatment with Everolimus or BEZ235 (10nmol/L and 100nmol/L) leads to a reduction in expression of the active form of the key downstream target proteins (pS6, pmTOR and pBad) of this pathway (SFigure 2F). To validate our AKT/mTOR array data, we performed western blot analysis of pS6 on protein extracts collected from 3D spheroids of Ishikawa cells that had been treated with Everolimus or BEZ235. Both inhibitors efficiently decreased the levels of phosphorylated form of S6 protein (SFigure 2G). Collectively, these findings showed that suppression of mTOR signaling decreased viability and proliferation of endometrial cancer cells.

## DISCUSSION

Elevated mTOR activity as seen with increased expression of the pS6 protein is observed in aged and/or cancerous tissue samples [23, 28]. Studies from various independent groups have confirmed that pharmacological or genetic suppression of mTOR signaling extends lifespan and healthspan in yeast, worms, flies, and rodents [12, 29, 30]. Rapamycin treatment suppresses tumorigenesis and extends maximal lifespan in cancer-prone mice [15, 17, 18, 31–33]. Rapamycin is safe for human use as it is routinely administrated in organ transplant patients without any major side effects [34]. Given dysregulated mTOR signaling is involved in the pathogenesis of many diseases, rapamycin is not only a promising anti-aging drug but could also be effective against other diseases such as cardiovascular and metabolic disorders, infertility, and cancer.

In young mice, mTOR signaling is required for estrogen-mediated growth of endometrial cells, and dysregulated mTOR signaling leads to female infertility due to defects in ovarian, oviductal, and endometrial functions [35–39]. Rapamycin treatment of young rats (5mg/kg i.p. on alternate days for 10wks) increases the ovarian follicular reserve [40], suggesting that treatment with this drug might prolong the reproductive lifespan, delay menopause, and improve reproductive fitness. However, all the rapamycin-rats displayed irregular estrous cycle and were unable to get pregnant [40]. It is now clear that several of the side effects of long-term treatment of rapamycin are due to disruption of mTORC2 signaling [13]. Because mTORC2 signaling is essential for follicular survival and endometrial development [41], it is likely that some of the reproductive functional defects observed after rapamycin treatment are due, at least in part, to altered mTORC2 signaling. Recently, two independent studies have shown that intermittent dosing of rapamycin delivers many of the benefits of rapamycin treatment on lifespan and healthspan with minimum negative effects on glucose homeostasis and immune system [42, 43]. It will be an interest of our future studies to examine if these new treatment regimens will have similar beneficial effects on reproductive functions of mice. In this study, we have shown that hyperactive mTOR signaling is present in the hyperplastic endometrium of aged women and mice, and inhibition of this pathway leads to suppression of endometrial lesions in mice.

Endometrial hyperplasia is commonly observed in postmenopausal women and aged mice [9, 10, 21]. In some of these women and mice (2%) these hyperplastic lesions progress to endometrial cancer [9, 10, 21]. The molecular mechanisms involved in the development of endometrial hyperplasia and its progression to cancer are currently unclear. Balanced estrogen and progesterone actions on endometrium are essential for endometrial proliferation and differentiation. It is well established that unopposed estrogen signaling leads to endometrial hyperplasia and cancer [44]. DNA and protein synthesis in response to estrogen is mediated through mTOR signaling in human and mouse endometrial cells [36], suggesting that mTOR signaling may mediate unopposed estrogen signaling in endometrial diseases, such as endometrial hyperplasia/cancer and adenomyosis. In this study, we have provided evidence that upregulation of mTOR signaling occurs in postmenopausal hyperplastic endometrium compared to postmenopausal normal controls. Genetic suppression by overexpressing the *Pten* gene and the pharmacological inhibition using rapamycin or rapalogs suppress the growth of human and mouse endometrial cells. Our findings suggest that targeting of the mTOR signaling pathway might be a viable strategy for treating age-related endometrial diseases.

## MATERIALS AND METHODS

### Mouse genetics and husbandry

Mice used in the present study were housed under standard animal housing conditions. All procedures for mouse experimentation were approved by the Animal Care and Ethics Committee at the University of Newcastle. Generation and characterization of Pten overexpressing mice (Pten^tg^) [45] and *Pten* heterozygous (Pten^+/−^) mice [46] are as previously described. Rapamycin treatment in mice has been described in a previous study [14]. Wild type C57BL/6, Pten^tg^ and Pten^+/−^ mice were aged and the female reproductive tissues were collected at regular intervals. Mice used in this study were specific pathogen free.

### Human endometrial tissue samples

Human endometrial tissue samples from 15 patients were obtained from the Hunter Cancer Tissue Biobank using a protocol approved by the Institutional Human Research Ethics Committee at the University of Newcastle.

### Cell lines, reagents and culture conditions

The human endometrial adenocarcinoma cell line, Ishikawa, was purchased from Sigma, (MO, USA) and validated with Short Tandem Repeat DNA profiling. Ishikawa cells were grown at 37°C in MEM medium supplemented with 5% fetal bovine serum (FBS), L-glutamine, and pencillin/streptomycin in a humidified atmosphere containing 5%CO_2_. Everolimus (RAD001) and NVP-BEZ235 were obtained from Selleckchem (Provided by Sapphire Biosciences, NSW, Australia). BD Matrigel^TM^ was used for the establishment of 3D cultures was obtained from BD Biosciences. Mycoplasma testing (MycoAlert^TM^ Plus Mycoplasma detection kit, Lonza, MD, USA) was conducted at regular intervals for the quality control of cell culture conditions.

### Histology and Immunohistochemistry (IHC)

For histological analyses, mouse uteri were fixed in 10% formalin solution (Sigma, MO, USA) overnight at 4°C and then transferred to 70% ethanol until processing. The fixed tissues were dehydrated in a series of graded ethanol, cleared in xylene, and embedded in paraffin wax. Embedded tissue samples were sectioned at 6µm and mounted on slides. Haematoxylin and eosin (H&E) staining and IHCs were performed using standard protocols described earlier [23]. Tissue sections were incubated overnight at 4°C with following primary antibodies: Phospho-S6 Ribosomal protein Ser235/236 (1:400; Cell Signalling Technologies, MA, USA), CK8 (Developmental Studies Hybridoma Bank, IA, USA). Biotinylated secondary antibodies (Jackson ImmunoResearch Labs, PA, USA or Thermo Fischer Scientific, Australia) were used followed by incubation with horseradish peroxidase-conjugated streptavidin (Thermo Fisher Scientific). Sections were then exposed to Diaminobenzidine (DAB, Sigma) to develop colour. Sections were counterstained with hematoxylin. Images were captured using Olympus DP72 microscope and the Aperio AT2 slide scanner (Leica Biosystems, Victoria, Australia). The gain and exposure time were set constant across tissue samples.

### Digital quantification of immunohistochemistry

Quantitative IHC analyses for pS6 staining were performed using the Halo™ image analysis platform (Indica Labs, New Mexico, USA). The pixel intensities of DAB staining were calculated using the Area Quantification algorithm. Pixel intensity values were then used to determine H-scores (calculated as the sum of 3 x % of pixels with strong staining + 2 x % of pixels with intermediate staining + 1x % pixels with weak staining).

### 3D cell viability and ATP assays

Cell viability in 3D cell culture was assessed using CellTiter-Glo^®^ Luminiscent cell viability assay (Promega, NSW, Australia) as per the manufacturer's instructions. Briefly, 5000 cells/well were seeded onto either 96-well ultra-low attachment plates or tissue culture plates coated with matrigel. Cells were allowed to grow for 24h for spheroid formation followed by desired drug treatments. Cell growth was monitored and images were photographed using JuLI^™^ Stage Real time cell history recorder (NanoEnTek Inc., USA). At 72h post- treatment, cells were incubated with CellTiter-Glo^®^ 3D Reagent for 30 minutes at room temperature and luminescence signal was recorded using the FLUOstar OPTIMA (BMG Labtech, VIC, Australia).

### Akt signaling antibody array and western blot analyses

Akt/mTOR signaling antibody array was performed as per manufacturer's instructions (Cell Signaling Technologies). Protein extracts from 3D culture of Ishikawa cells treated with different concentrations of Everolimus or BEZ235 were prepared in ice-cold radio-immunoprecipitation assay buffer (RIPA) supplemented with protease and phosphatase inhibitors. Equal amounts of protein were loaded and separated by 10% SDS-PAGE gel and thereafter transferred to nitrocellulose membrane. The membrane was blocked in 5% milk (w/v) in Tris-buffered saline/Tween 20 for 1 hr at room temperature and then incubated overnight at 4°C with rabbit mAb Phospho-S6 Ribosomal protein (1:2000 in 2.5 w/v BSA, 1xTBS, 0.1% Tween-20; Cell Signaling Technologies). This was followed by incubation with secondary horseradish peroxidase-conjugated anti-rabbit antibody (Jackson ImmunoResearch, West Grove, PA) for 1hr at RT. β-actin was used as a loading control.

### Statistical analysis

Statistical analyses were performed using GraphPad Prism 6.0 (Graphpad Software, San Diego, CA). Values are expressed as mean± SEM. The Student *t* test was used to calculate differences between the groups (N≥3/group), and *p* values ≤ 0.05 were considered statistically significant.

## SUPPLEMENTARY MATERIALS TABLES FIGURES


