# Supplementary material for: Overactive mTOR signaling leads to endometrial hyperplasia in aged women and mice
Source: Oncotarget. 2016 Dec 12;8(5):7265–75. doi: 10.18632/oncotarget.13919 (PMC5352319; doi:10.18632/oncotarget.13919)
Supplement: Supplementary file 1 [file oncotarget-08-7265-s001.pdf]

Overactive mTOR signaling leads to endometrial hyperplasia in aged women and mice

Supplementary Material

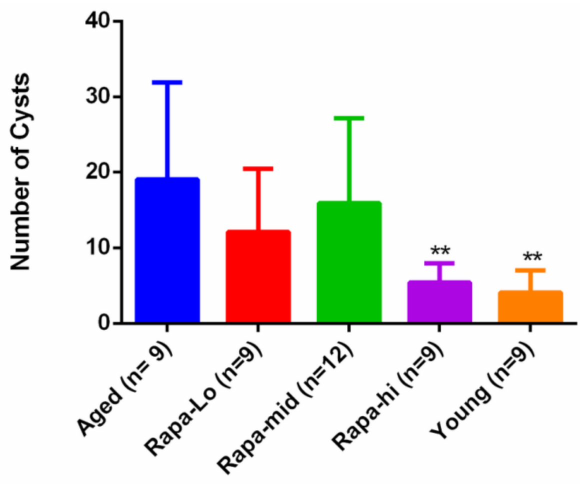

SFig. 1. Quantification of a number of epithelial cysts in uteri of different rapamycin treated groups and control.

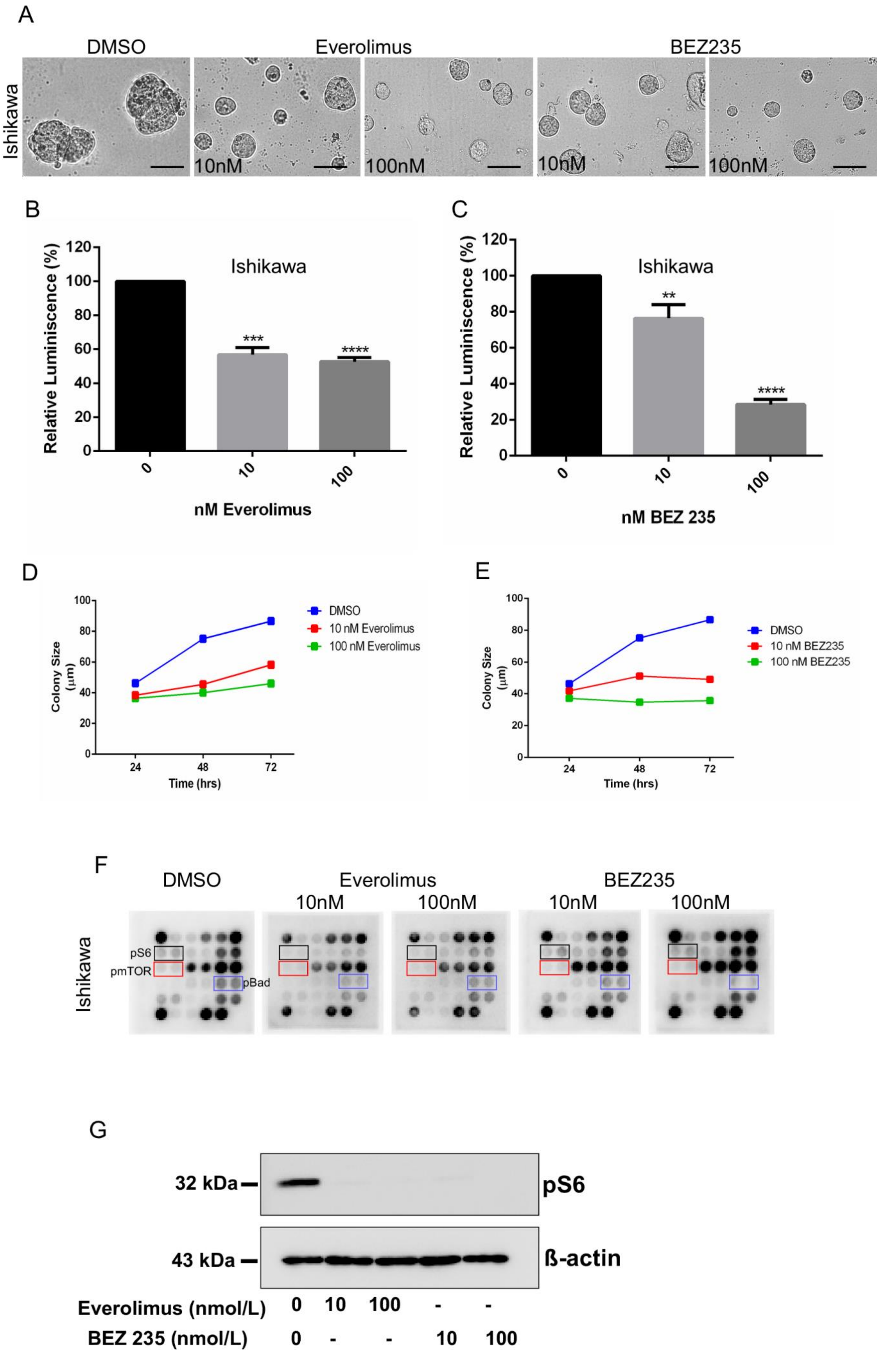
A
DMSO
Everolimus
BEZ235
Ishikawa
10nM
100nM
10nM
100nM
B
Ishikawa
Relative Luminiscence (%)
0
20
40
60
80
100
120
***
****
0
10
100
nM Everolimus
C
Ishikawa
Relative Luminiscence (%)
0
20
40
60
80
100
120
**
****
0
10
100
nM BEZ 235
D
Colony Size (μm)
0
20
40
60
80
100
24
48
72
Time (hrs)
DMSO
10 nM Everolimus
100 nM Everolimus
E
Colony Size (μm)
0
20
40
60
80
100
24
48
72
Time (hrs)
DMSO
10 nM BEZ235
100 nM BEZ235
F
DMSO
Everolimus
BEZ235
10nM
100nM
10nM
100nM
Ishikawa
pS6
pmTOR
pBad
G
32 kDa
pS6
43 kDa
ß-actin
Everolimus (nmol/L) 0 10 100 - -
BEZ 235 (nmol/L) 0 - - 10 100

SFig. 2. mTOR inhibitors suppress the growth of 3D spheroids of Pten-negative endometrial cancer cells. Representative pictures of Ishikawa cells cultured in 3D with increasing doses of mTOR inhibitors, Everolimus and BEZ235 (A). 72 hours after culture, cell viability was assessed using CellTiter-Glo® Luminiscent cell viability assay (B and C). Colony size for 3D spheroids was measured after 24h, 48h and 72h of treatment with mTOR inhibitors (D and E). The data shown are mean ±SEM of three individual experiments. $^{**}P<0.01$, $^{***}P<0.001$, $^{****}P<0.0001$, Student's *t*-test. Ishikawa cells in 3D culture were treated with DMSO control or increasing concentrations of Everolimus or BEZ235. 72 hours later, whole cell lysates were subjected to AKT/mTOR signaling antibody protein array (F). Whole cell lysates from Ishikawa spheroids treated with increasing doses of Everolimus and BEZ235 were subjected to western blot analysis for pS6 (G). The data shown are representative of three individual western blot analyses. β-actin was used as a loading control.
